# Court-Mandated Treatment for Convicted Drinking Drivers

**Published:** 2006

**Authors:** Patricia L. Dill, Elisabeth Wells-Parker

**Affiliations:** Patricia L. Dill, Ph.D., is an assistant research professor, and Elisabeth Wells-Parker, Ph.D., is a professor, both at the Social Science Research Center, Mississippi State University, Mississippi State, Mississippi

**Keywords:** health services research, AOD (alcohol and other drug) offense, AOD offender, societal AODR (AOD-related) problems, drinking and driving, AOD use and driving, DWI (driving while intoxicated) laws, DWI arrest, impaired driver, traffic accident, mandatory treatment, mandatory participation, mandatory screening programs, AODU (AOD use) treatment method, treatment program, intervention, cost-effectiveness

## Abstract

Court-mandated treatment, which requires offenders convicted of alcohol or other drug–related crimes to participate in treatment for their substance abuse problems or face legal consequences, has long been a component of sanctioning for driving under the influence (DUI) and is a primary path of entry into alcoholism treatment for many people with problem drinking. Several issues are relevant to mandated treatment: screening, assessment and referral, effectiveness, DUI events as opportunities for intervention, brief interventions for offenders outside of mandated treatment, and cost-effectiveness of mandated treatment. Treatment effectiveness depends to some extent on offenders’ motivation to participate, and offenders may resist treatment when their participation is coerced. Types of treatment such as motivational enhancement therapy may prove cost-effective with these involuntary participants. More research is needed into the changing DUI population, impaired driving and multidrug use, and new technologies for monitoring DUI offenders.

Offenders in the criminal justice system who are charged with crimes related to alcohol use (e.g., public drunkenness, driving under the influence [DUI], and underage drinking) can be sentenced to participate in some form of treatment for alcohol problems. This may consist of formal treatment as well as other rehabilitative interventions designed to address problem drinking and its harmful consequences. Court-mandated treatment remains a primary route by which many people enter alcoholism treatment (Weisner et al. 2002) (see textbox, p. 42).

The terms *mandated treatment* and *coercion* often are used interchangeably (Farabee et al. 1998). Mandated treatment is accompanied by “threats of legal consequences if individuals refuse to comply with a referral to treatment” (Polcin and Greenfield 2003, p. 650). Offender perceptions of the likelihood and severity of these sanctions (e.g., jail time or house arrest) are critical determinants of whether these offenders comply with the treatment mandate (Cavaiola and Wuth 2002).

Court-mandated treatment to reduce drinking and driving and treat alcohol problems has been a common element of the sanctioning process, especially for DUI offenders, for several decades. This article focuses on mandated treatment for DUI offenders, who account for a large proportion of those legally required to attend treatment for problems arising specifically from alcohol use (Cavaiola and Wuth 2002; Weisner 1990). The following sections examine forms of mandated treatment; screening, assessment, and referral; the effectiveness of mandated treatment, including treatment matching; DUI events as opportunities for intervention; and brief interventions for offenders outside of mandated treatment. In addition, this article discusses treatment cost-effectiveness and access as well as future research needs and challenges. An exhaustive discussion of research needs for improving alcohol interventions, including treatment, with impaired drivers mandated to treatment in the legal system is beyond the scope of this article.

## Forms of Mandated Treatment

Mandated interventions for DUI offenders vary in intensity, frequency, and duration, ranging from relatively brief one- or two-session interventions, to multicomponent programs implemented over the course of weeks or months, to inpatient care with lengthy aftercare (Wells-Parker et al. 1995). Treatment referrals may involve several components because DUI offenders are diverse, both in terms of level of alcohol abuse and other characteristics, such as comorbid conditions, that may increase their risk of repeating their offense or becoming involved in a crash (Wells-Parker and Popkin 1994).

DUI offenders who have been mandated to treatment by the courts participate in a wide variety of alcoholism treatment programs (Cavaiola and Wuth 2002; Wells-Parker et al. 1995). Mandated interventions for DUI offenders can include generic alcoholism treatment programs offered in local communities, referral to groups such as Alcoholics Anonymous (AA), and strategies that specifically aim to reduce drinking and driving, such as education programs, supervised probation, and presentations by injured survivors or families of victims killed in alcohol-related crashes (i.e., victim impact panels). Mandated interventions often include supervised probation and other forms of supervision and monitoring as well. In addition to monitoring, these programs can provide supportive contact and assistance with problems that could contribute to the risk of driving while impaired (Wells-Parker et al. 1995).

In the early years of DUI programs, traditional educational programs that focused on teaching offenders about how alcohol impairs driving were based on the premise that most DUI offenders were social drinkers who had too much to drink on one occasion. However, a large body of evidence (Cavaiola and Wuth 2002; Wells-Parker et al. 1995) shows that convicted offenders have a range of drinking problems, as well as other problems that contribute to crash risk, and frequently are at high risk of crashes even when not impaired (Cavaiola and Wuth 2002). As a result of this research, most educational and specialized programs have moved from a primarily didactic approach to interventions with specific protocols (Hon 2003). Specialized interventions are being developed to reduce alcohol-impaired driving and address alcohol problems and other comorbid conditions that frequently occur among DUI offenders (Cavaiola and Wuth 2002; Hon 2003; Wells-Parker and Williams 2002).

**At a Glance**

**Criminal Justice System Referrals to Substance Abuse Treatment**

- In 2002, alcohol was the most frequently reported primary substance of abuse^1^ among all substance abuse treatment admissions.
- The criminal justice system was the principal source of referral for 36 percent of all substance abuse treatment admissions in 2002 (655,000 referrals out of a total of 1.9 million admissions).
- Compared with people referred to substance abuse treatment from other sources, people referred by the criminal justice system were more likely to:
  – Report alcohol as the primary substance of abuse.
  – Be younger than age 25.
  – Report that they had never been treated for substance abuse problems elsewhere.
  – Be treated in ambulatory treatment settings.

SOURCE: Substance Abuse and Mental Health Services Administration (SAMHSA). *The DASIS Report: Substance Abuse Treatment Admissions Referred by the Criminal Justice System: 2002*. Washington, DC: Office of Applied Studies, SAMHSA, July 30, 2004.1The primary substance of abuse is the main substance reported at the time of admission.

## Screening, Assessment, and Referral

DUI offenders mandated by the courts to receive intervention and treatment often are evaluated in terms of their future risk for impaired driving and crash involvement and for any personal problems or circumstances that may need to be addressed during intervention and treatment. The term *screening* typically is used to describe a less extensive evaluation performed early in the process, possibly before a referral is made or shortly thereafter, and tends to focus on determining the offender’s risk level for impaired driving and the extent of alcohol problems. Screening results often are used to make decisions about what type of intervention is mandated. The term *assessment* typically is used to refer to a more extensive evaluation that is conducted later, often just before or upon entry into intervention and treatment. Assessment results frequently are used to guide decisions about how to intervene and treat the offender and how long or intense the treatment will be. The ultimate goal of extensive assessment is to match the offender to the most appropriate intervention and treatment according to his or her specific circumstances. Often assessment is integrated into the intervention in order to guide the process and to assure that the offender’s problems are being addressed.

The quality of information provided by either a screening or an assessment is an important part of the intervention and treatment process. One concern about court-referred assessment of alcohol and other substance use problems is that offenders may minimize their involvement with alcohol if they believe their answers could result in harsher sentencing or more intensive treatment (Lapham et al. 2002). In addition, a conflict of interest may arise when the same entity that will provide treatment conducts the assessment, which determines treatment length and cost.

To address these concerns, valid and reliable screening and assessment processes that are not dependent on subjective and unvalidated judgments of assessors ultimately need to be developed. Standards for validating the screening and assessment procedures that inform referral and treatment decisions are critical to ensuring successful outcomes for clients. (For a review of the technical issues surrounding the development of valid and reliable screening and assessment tools and processes for use with mandated populations, and for standards for validating these tools, see Anderson and colleagues [2000].)

## Effectiveness of Mandated Treatment

Systematic research on mandated treatment for DUI offenders since the early 1980s (Mann et al. 1994; Wells-Parker and Williams 2004) has provided a relatively clear picture of the effectiveness of this treatment as well as its limitations. In general, research has consistently shown that treatment has a modest effect on reducing drinking–driving and alcohol-impaired crashes among offenders who are mandated to attend and who actually receive the intervention (Wells-Parker and Williams 2002).

A meta-analysis of studies of the effectiveness of treatment and intervention with DUI offenders revealed several reliable patterns (Wells-Parker et al. 1995). An examination of crashes and DUI events over several years showed that alcohol-specific interventions and alcoholism treatment were better at reducing alcohol-related driving and crashes than interventions which were not alcohol specific. However, nonspecific interventions—such as revoking drivers’ licenses—were better at reducing all types of crashes (including crashes that did not involve alcohol), probably because they reduce overall driving exposure. Thus, the best strategy is to combine alcohol-related interventions and treatment with licensing actions to reduce impaired driving and crashes in general among DUI offenders who, as a group, are known to be high-risk drivers even when not impaired (Donovan et al. 1988; Donovan et al. 1985). Although the meta-analysis was conducted in 1992, more recent studies generally have confirmed the results (Hon 2003). Combining treatment with nontreatment sanctions that prevent offenders from drinking and driving (e.g., license revocation and alcohol ignition interlocks, which require the driver to pass an alcohol breath test before starting a car) also reduces the public’s risk while offenders are receiving treatment.

Findings from the meta-analysis did not reveal a consistent pattern of results for outcome measures related to drinking problem severity or other non-traffic-related outcomes because most studies focused on recidivism and crashes (Wells-Parker 1994; Wells-Parker et al. 1995). Results of one long-term study in which offenders were randomly assigned to receive treatment suggested that mandated interventions may have benefits beyond the traffic safety arena. In this study (Mann et al. 1994), offenders who received treatment had lower mortality rates after several years than did members of a comparable group who did not receive treatment.

Because only a few rigorous methodological studies have evaluated specific interventions, it was not possible in the meta-analysis to draw broadly substantiated conclusions about most treatment and intervention strategies used with DUI offenders (Wells-Parker 1994; Wells-Parker et al. 1995). The most effective strategy, which had substantial support from rigorously conducted studies, combined education and treatment. The treatment component included counseling or psychotherapy and supportive followup such as probation. Program intensity or length did not entirely explain the superiority of combination programs. Combining strategies may be more effective, regardless of treatment length or intensity, because DUI offenders have diverse and complex problems, and offering varied approaches may help to address this range of problems. Using a combination of strategies also increases the likelihood that at least one of the strategies will be effective for a particular offender.

Some methods may have little effect by themselves but could be useful in combination with other strategies. In the meta-analysis (Wells-Parker et al. 1995), only two reviewed studies evaluated direct court referral to AA, and those studies did not show that mandatory AA participation alone had a beneficial effect on recidivism. However, other studies suggested that combinations incorporating AA attendance often were effective.

A meta-analysis of controlled studies of the effectiveness of AA (Kownacki and Shadish 1999) found that randomized studies, but not nonrandomized studies, of AA alone produced more negative outcomes than no treatment at all. Also, effects of AA-based residential treatment programs were much smaller in randomized studies compared with nonrandomized studies, but the small number of randomized studies resulted in nonsignificant differences when compared with alternative residential treatment. The randomized studies contained several samples of coerced participants, whereas the nonrandomized studies used only samples of voluntary participants, indicating that forced AA attendance may be worse than no treatment (Kownacki and Shadish 1999).

Offenders increasingly are required to attend victim impact panels (VIPs), sometimes in addition to remedial interventions or treatment. However, several rigorous studies have failed to show positive effects of VIPs on recidivism rates (Wells-Parker 2004). Currently, investigators do not know what factors may influence whether VIPs are effective or not in reducing recidivism. Clearly, mandating this form of intervention should await a more thorough evaluation of the effects of VIPs.

### Matching Offenders to the Most Effective Treatment Strategy

In addition to alcohol abuse, many DUI offenders have individual characteristics (such as a propensity for risktaking in general and, specifically, a tendency to take risks while driving [Donovan et al. 1988; Donovan et al. 1985]) or comorbid conditions (such as depression) that either are likely to contribute to harmful consequences associated with alcohol use (e.g., drinking and driving) or must be considered if treatment is to be successful (Cavaiola and Wuth 2002; Wells-Parker et al. 1995). Research that attempts to identify the most effective treatment based on a person’s individual characteristics (i.e., treatment-matching) has been an important issue for treatment research (National Institute on Alcohol Abuse and Alcoholism [NIAAA] 2000).

**Difficulties in Researching Court-Mandated Treatment**

The study of mandated treatment has several inherent research challenges that are in need of creative solutions:

- ***State-to-State program variations***. Although general guidelines for the assessment and referral of DUI offenders have been developed (National Highway Traffic Safety Administration [NHTSA] and National Institute on Alcohol Abuse and Alcoholism [NIAAA] 1996), assessment, referral, and intervention systems vary widely across jurisdictions in the United States (Cavaiola and Wuth 2002). As a result, mandated treatment requirements also vary (Cavaiola and Wuth 2002; Wells-Parker 1994). Furthermore, the strength of the mandate to receive treatment is weakened by wide variations in the frequency and timeliness of imposing contingent sanctions (e.g., jail time or house arrest) on those who fail to attend or complete treatment (Wells-Parker 1994). These inconsistencies make studying mandated treatment difficult.
- ***Difficulty of using random assignment***. The gold standard of research design, random assignment, is extremely difficult to achieve in court systems, where it conflicts with usual procedures.
- ***Problems with outcome measures***. Defining the goals of mandated treatment and determining how best to measure them can be challenging. Is the intended goal of mandated treatment for drinking–driving offenders reduced recidivism or reduced alcohol consumption and related problems? Treating alcohol problems and reducing impaired driving/crash risk, though interrelated, represent different goals (Wells-Parker and Williams 2004). Using recidivism and crash involvement as outcome measures has some ecological validity (i.e., the findings reflect reality), but these measures have short-comings as surrogates for drinking and driving, such as infrequent occurrence (i.e., most drinking-and-driving events result in neither an arrest nor a crash), possible biases, and when based on official records for local or State sources, incomplete information (Wing 2004). Other measures, such as self-report or collateral reports of drinking and drinking-and-driving behavior, could have some advantages but are problematic with this population because offenders are difficult to track over periods of time and because, as in the assessment phase, offenders being tracked as well as their significant others may be motivated to minimize problem behaviors. Technological innovations such as interlocks and off-site monitoring offer interesting new possibilities for tracking drinking-and-driving outcomes but may pose their own challenges.

—Patricia L. Dill and Elisabeth Wells-Parker

ReferencesCavaiolaAWuthCAssessment and Treatment of the DUI OffenderNew YorkHaworth2002National Highway Traffic Administration (NHTSA) and National Institute on Alcohol Abuse and Alcoholism (NIAAA)A Guide to Sentencing DUI OffendersDOT HS–808–365Washington DCNHTSA and NIAAA1996Wells-ParkerEMandated treatment: Lessons from research with drinking and driving offendersAlcohol Health & Research World1843023061994PMC687644331798088Wells-ParkerEWilliamsMInterpreting research for practice: A challenge for evidence-based assessment and intervention with DWI offenders. Book reviews of *Assessment and Treatment of the DWI Offender* by A. Cavaiola and C. WuthContemporary Psychology: APA Review of Books491611642004WingSA review of practice and research on alcohol-impaired drivingFrontlines: Linking Alcohol Services Research and PracticeWashington, DCNIAAA9200438

Although many treatment-matching studies may include DUI offenders, most have not focused on DUI offenders as a distinct group (Wells-Parker et al. 1995). For example, a large treatment-matching study (Project MATCH), which did not focus specifically on mandated offenders (Project MATCH Research Group 1997), found that people in alcoholism treatment who were angry benefited most from motivational enhancement therapy (NIAAA 2000). This form of therapy is designed specifically to lower resistance to treatment and enhance motivation to change (Project MATCH Research Group 1997). Participants without good support systems for drinking cessation and changing problem behaviors fared best in a 12-step program, in which AA attendance was more likely (NIAAA 2000). People with low levels of psychiatric severity also fared best after 12-step facilitation treatment (NIAAA 2000). Because many DUI offenders entering mandated programs are angry about their arrest and sentencing, nonconfrontational strategies that are designed to enhance motivation may be especially appropriate. In addition, some offenders lack social support networks that discourage drinking as well as drinking and driving (Cavaiola and Wuth 2002). Strategies that encourage, but do not mandate, attendance at AA or other support groups are likely to be appropriate for these offenders also.

A recent study (Wells-Parker and Williams 2002) examined the effects of adding a brief individual intervention component to an existing court-mandated group intervention program for first-time DUI offenders. These researchers were particularly interested in which offenders benefited most from the additional supportive counseling. Approximately 4,000 first-time DUI offenders were randomly assigned to either a standard first-offender program or to the standard program plus the brief counseling component (the combination program).

In the standard program, offenders were exposed to cognitive-behavioral and motivational techniques in groups and through homework assignments and some education concerning the effects of alcohol and other drugs on health and behavior. The combined intervention added two 20-minute sessions of supportive counseling that provided individual feedback concerning problems such as feelings of sadness; these additional sessions were designed to enhance motivation and the confidence to change behavior.

The recidivism rate for offenders who did not report depressed mood was similar for the two programs. However, offenders who reported being depressed and who received the combination program had recidivism rates that were 35 percent lower than those of depressed offenders who received the standard program only. Results suggested that depressed offenders initially were more likely to recognize that they had a drinking problem and needed to change, and were more likely to try to change, than those not reporting depression, but the depressed offenders also were less confident in their ability to change. The supportive counseling may have been especially appropriate for depressed offenders who wanted to change their behavior but lacked confidence to do so. For some DUI offenders, depression may be an indicator of readiness to change, but a lack of confidence in their ability to change results in a feeling of hopelessness. Brief supportive counseling may allow the offender to explore and overcome this barrier.

Because many offenders, especially those with more severe alcohol problems, are depressed (Cavaiola and Wuth 2002; Wells-Parker and Williams 2002), it is important to acquire a better understanding of how to target appropriate interventions to depressed offenders. For example, brief supportive counseling that focuses on changing alcohol-related problem behavior seems to reduce recidivism. It is not known, however, whether an intervention that specifically targets depression would be equally or more effective, not only in managing depression but also in supporting change in alcohol-related problem behavior among mandated offenders. More research also needs to focus on the effectiveness of treating other comorbid psychiatric conditions that DUI offenders frequently have, such as anxiety disorders, antisocial personality disorder, mood disorders, and post-traumatic stress disorder (C’de Baca et al. 2004).

## A DUI Event as a Window of Opportunity to Encourage Behavior Change

Recent studies suggest that levels of motivation and readiness to change are critical to the success of mandated treatment, and assessment and treatment should include components which target these issues (Farabee et al. 1998). A DUI arrest and conviction may represent an opportunity to increase motivation by helping an offender recognize his or her problem with drinking and its consequences. For example, most first-time offenders who entered a DUI program acknowledged that they needed to change both their drinking and their drinking-and-driving behavior, and indicated that they were trying to do so (Wells-Parker and Williams 2002). To take advantage of the window of opportunity that may be created by the DUI event, strategies designed to be nonconfrontational and to support motivation to change and the development of realistic change plans may be especially useful as components of DUI programs. These strategies also may be useful in increasing motivation for additional treatment when problems are severe.

## Brief Interventions With DUI Offenders Outside of the Mandated Treatment System

Many people who drink, drive, and are involved in traffic crashes are treated for injuries but do not enter the criminal justice system (Dill et al. 2004). A crash, therefore, especially one resulting in injuries, may offer another opportunity to motivate change. Recent efforts have focused on intervening with alcohol-positive drivers who are injured in crashes and are treated for those injuries in medical settings such as emergency departments or trauma centers (Dill et al. 2004).

Brief interventions often consist of only one or two short sessions, which are compatible with busy medical settings. These interventions generally have several components, including individualized feedback from a short screening, brief advice, and specially adapted counseling strategies appropriate for short sessions. These types of interventions have been offered to alcohol-positive drivers treated in emergency departments and trauma settings and have been found effective in reducing drinking and driving and other harmful behaviors (Dill et al. 2004), as well as mortality (Cuijpers et al. 2004). Brief interventions can be offered in a broad range of settings, are cost-effective, and may be used along with other rehabilitative modalities to enhance the motivation and self-confidence to change drinking-and-driving behavior.

## Treatment: Cost-Effectiveness and Access

Research has not specifically examined the cost-effectiveness of mandated treatment for drinking and driving. However, considering that alcohol was a factor in 41 percent of U.S. traffic deaths in 2002 (Hingson and Winter 2003), and that the U.S. economic costs related to alcohol use problems (not counting the costs of prevention and treatment) were about $177 billion in 1998 (NIAAA 2000), cost-effective treatments are imperative.

It should be noted that the degree of cost-effectiveness depends on the treatment outcome considered—such as reduced health care costs, legal costs, or work-related costs (NIAAA 2000; Sindelar et al. 2004). When considering reduced health care costs, studies of the cost-effectiveness of alcoholism treatment in general suggest that many treatment modalities are cost-effective; however, more expensive treatments do not necessarily yield better outcomes (NIAAA 2000). Outpatient treatment, when appropriate, is considered the most cost-effective measure, but people with heavy alcohol dependence may require inpatient services to reap the most benefit relative to cost. In addition, studies have found that reducing inpatient treatment from 28 days to 21 days yields similar outcomes at a more cost-effective rate (NIAAA 2000).

Research is needed to determine the cost-effectiveness of mandated treatment. Overall treatment effectiveness may be influenced by offenders’ levels of motivation and by hostility resulting from coercion. Certain types of treatment, such as motivational enhancement therapy, may prove to be more cost-effective in these involuntary circumstances, as demonstrated with findings from Project MATCH (NIAAA 2000).

### Treatment Costs and Insurance Reimbursement

Compared with the previously mentioned $177 billion that alcohol use problems cost the U.S. economy in 1998, this country spent only $7.5 billion on treatment (NIAAA 2000). Thus, the development of cost-effective alcoholism intervention and treatment may represent an unrealized opportunity to lessen the negative consequences of alcohol problems nationwide. However, third-party payers are allowed by law to refuse coverage for court-ordered treatment (Wing 2004). Often, even if coverage is available, it is insufficient for a clinically meaningful length and intensity of treatment (Wing 2004). Clearly, current policy deters potential third-party coverage for mandated treatment, although such coverage may be available if entry into treatment was voluntary.

## Future Research Needs

As noted in this review, many questions concerning mandated treatment remain unanswered. Emerging issues related to changing DUI offender populations, multidrug use by impaired drivers, and new technologies for monitoring DUI offenders require rigorous study to determine how to provide more effective court-referred treatments.

### Changes in the DUI Offender Population

The characteristics of the DUI offender population have been changing in response to stricter laws, such as zero-tolerance laws for underage drinkers (which set the legal blood alcohol limit for drivers younger than age 21 at 0.00 or 0.02 percent). The DUI offender population also reflects changes in the demographics of the general population (such as increasing ethnic diversity). Courts will need varied intervention options to meet the needs of this changing population.

#### Youth

The National Highway Traffic Safety Administration (NHTSA) estimated that in 1998, drivers ages 16–20 drove 12 million times within 2 hours of drinking (Hingson et al. 2004), and the average calculated blood alcohol concentration (BAC) for this age group was 0.10 percent, or more than three times the estimated average BAC of drivers of all ages who drove after drinking (Hingson et al. 2004). After the advent of zero-tolerance laws, studies showed a 19-percent reduction in the proportion of underage drinkers who drove after any drinking, and a 23-percent reduction in driving after consuming five or more drinks (Hingson et al. 2004).

Even with the significant reduction in the proportion of underage drinking drivers, many young people continue to drink and drive (Hingson et al. 2004). With zero-tolerance laws, drinking–driving youth who might otherwise have avoided criminal sanctions can be mandated to treatment.

Few studies have examined the effectiveness of interventions specifically to reduce drinking and driving by adolescents; most studies have examined adolescent problem drinking. A recent review of strategies to reduce problem drinking among college students found that information-based and values-clarification programs were not effective, while some skills-based interventions (e.g., self-monitoring skills) did reduce alcohol consumption effectively (Larimer and Cronce 2002). Although individually oriented interventions such as risk skills training, designed to help youth recognize and reduce risky behavior, as well as brief interventions, have been found to reduce adolescent drinking and driving, research is needed to determine the most effective treatment modalities for underage drinking-and-driving offenders mandated to treatment. Would mandated group interventions specifically targeted to adjudicated underage drinking drivers be more effective than mandated group interventions that include all age groups?

Programs that address important youth issues related to alcohol and drinking-and-driving behavior by young people in peer group settings may be efficacious. However, group modalities, often typical in interventions for first-time DUI offenders, may be counterproductive for groups consisting solely of adolescents. Studies have shown that putting adolescents who have high levels of a problem behavior together for group intervention actually can escalate the problem behavior (Poulin et al. 2001). On the other hand, underage offenders in a mixed-age group could recognize the difficulties experienced by older, chronic drinkers as negative consequences to avoid and could become more motivated to change their behavior. Definitive research is needed to resolve this issue.

#### Racial and Ethnic Diversity

Studies suggest that the environment of drinking, reasons for drinking, and levels of alcohol consumption vary for Hispanics, African Americans, and non-Hispanic Whites (Arroyo et al. 1998; Caetano and Raspberry 2001; Gil et al. 2004). Research is needed to determine if culturally specific mandated treatment would be more effective for ethnic and racial minorities and whether such treatment would be feasible and cost-effective. Appropriate interventions also are needed for non-English-speaking populations.

### Multidrug Use

People often abuse alcohol along with other drugs, and driving while impaired as a result of multidrug use is becoming increasingly recognized as a major factor in vehicular crashes (Ogden and Moskowitz 2004). Determining whether a person has used psychoactive drugs such as cannabis, opioids, cocaine, and psychoactive medications in combination with alcohol is difficult logistically because it requires an analysis of body fluids (Ogden and Moskowitz 2004).

People who abuse both alcohol and other drugs often are treated in programs that are not specific to alcohol. Recent data suggest that approximately 36 percent of referrals to substance abuse treatment programs are the result of mandates from the criminal justice system (Substance Abuse and Mental Health Services Administration [SAMHSA] 2004), and some jurisdictions routinely offer treatment for offenders charged with substance-abuse-related crimes as an alternative to prison (Weisner et al. 2002). Research is needed to broaden the scope of mandated treatment for impaired driving to include interventions for multidrug users (McCarty 2004).

### Integration of Treatment With New Technology and Specialized DUI Courts

New technological developments, such as alcohol ignition interlocks and electronic monitoring devices, which allow home detention and remote BAC monitoring (Voas 2004), may provide new sanctioning options that can be integrated effectively with more traditional intervention and treatment modalities to reduce DUI recidivism. For example, researchers are investigating the possibility of using BAC data from alcohol interlock systems to monitor offenders’ alcohol use as part of treatment (Voas 2004). Such data can be valuable to treatment providers, but making the information routinely available may require procedural changes. Technological monitoring would allow courts to set performance-based sanctions and treatment goals. Research shows that the breath test results recorded on interlocks predict future recidivism (Marques et al. 2003). Therefore, this objective record of drinking may be used to measure problem drinking status and provide a means for tailoring treatment to individual offenders and for improving the overall efficiency of mandated treatment.

DUI courts, which are patterned after specialized courts for drug offenders, offer extensive monitoring of DUI offenders’ driving behavior and alcohol use (Voas 2004). These programs, which currently are being evaluated, may offer an alternative to traditional adjudication strategies, depending on the outcome of the ongoing evaluations. Operating DUI courts can be expensive, and it will be important to determine if adopting new technologies such as remote electronic BAC monitoring can help reduce costs (Voas 2004).

## Summary

Court-mandated treatment to reduce drinking and driving and treat alcohol problems has been a common element of the sanctioning process for several decades. Although research has identified some effective mandated treatment modalities, knowledge gaps remain, and further research is needed to understand how best to intervene. Researchers will need to make particular effort to overcome several research challenges inherent to this field of study.
